# Independent Agencies and Research Program Performance

**DOI:** 10.3389/frma.2022.856862

**Published:** 2022-05-24

**Authors:** Charles N. W. Keckler

**Affiliations:** Schar School of Policy and Government, George Mason University, Fairfax, VA, United States

**Keywords:** government performance, research metrics, executive branch, independent agencies, federal programs, Bush Administration, Program Assessment Rating Tool

## Abstract

Combining performance data from the Bush Administration's Program Assessment Rating Tool (PART) initiative with measures of organizational independence, I examine whether insulated and plural leadership structures are consequential for the outcomes of the federal programs administered by them. Using regression modeling and controlling for program type, I find that embedding programs in independent agencies is positively and significantly related to ratings of program performance. The effects of independent commissions appear mediated in these models by their positive association with the PART scores given to certain program types, notably research programs. These results are problematic for any global attribution of greater effectiveness to executive agencies under single-headed control and closer presidential direction.

## Introduction

There are two basic ways to carry out the executive functions of government, but no one knows which way is better. Public sector organizations, with some variations, tend to fall into two types: most commonly, administrative agencies are headed by a single administrator accountable and tied politically to a chief executive. The principal alternative form to this “ordinary” executive department is an “independent agency” usually headed by a plural leadership group such as a commission, which is often mulitpartisan by design and insulated from the influence of an elected head of government (Lewis and Selin, 2013; O'Connell, 2013, p. 97; for international analogs, see Gilardi and Maggetti, 2011). The key distinction in the American federal context, on which i concentrate henceforth, is in the agency's governance structure, which to varying degrees is under the indirect authority of Congress and direct authority of the President, and which defines for the agency the legal and political interface between democratic control and the implementing bureaucracy (Moe, 1995). The insulation of agency leadership from direction by the head of the executive branch, most notably protection from being removed in the case of presidential policy disagreement, together with agency financial autonomy from the legislature, broadly if incompletely define the “independence” that *de facto* and *de jure* distinguish the American multimember independent commission (MIC) (Datla and Revesz, 2012; Selin, 2015). Although itself having some level of variation as discussed below, this organizational type usually is seen as forming one half of a bureaucratic dichotomy with single-headed executive departments (SHED) (Lewis and Selin, 2013, p. 10; Breger and Edles, 2015, p. 4). A natural inquiry that follows from this typology, but one difficult to answer, relates to what—if any—functional differences in government operations follow from these structural distinctions (Seidman, 1998, p. 186). Clarity on this fundamental question is hard to achieve in large part because the necessary agency comparisons are hampered by an inability to “measure US government performance systematically across different contexts” (Gallo and Lewis, 2012).

Despite the formidable empirical challenges, pursuing this question is not only of theoretical but also of practical interest, because policy choices continue to be made about the appropriate institutional framework for government activities. Congress regularly creates organizations of both types—about one-third of them independent and two-thirds of them not—although political rather than efficiency considerations appear to drive these decisions (Lewis, 2004).

Even more often, Congress must choose whether to assign new programs and activities to different types of agencies, a choice also typically analyzed in political terms (Lavertu, 2013). Perhaps it would be normatively ideal for Congress to make institutional choices of the environment most conducive for the efficient and effective implementation of specific executive activities, if it had the evidence to do so. However, even if politics continued to predominate, as a realistic appraisal of legislative incentives suggests, we should seek the costs and benefits (in terms of performance effects) that these political choices represent. Thus, Krause's useful formulation of the central question remains pertinent, if unanswered: “Does legislative delegation to an insulated agency (e.g., independent commission) provide superior performance vis-à-vis a less insulated agency under the direct aegis of the executive branch? If not, why not?” (Krause, 2010).

In order to make progress on this problem, the focus of the Article is an attempt to detect performance differences between these two broad types of agencies (independent/non-independent, or as I sometimes refer to them, MIC/SHED). After a brief consideration of the theoretical context of the study, I make use of a well-known dataset of agency information derived from the Program Assessment Rating Tool (PART) implemented in the George W. Bush Administration^1^. This remains one of the few systematic sources of data on core agency functions which is comparable across both agencies and agency types. While far from infallible in its judgments of program performance and therefore to be used with caution, PART has provided a rich source of raw material for testing the hypotheses of public administration scholars (Moynihan, 2013; Kroll and Moynihan, 2021).

Another research advantage of the PART information collection, beyond its relative comprehensiveness, is that it partitions federal programs into seven different categories, permitting a more specific (if still high-level) comparison of how well broad kinds of activities perform in different types of agencies. This is a first step toward understanding the consequences of legislative issue delegation—which has the potential to be a more nuanced and practical inquiry than would a simple attempt to determine which type of agency is “better.” In the process of analyzing this data, it soon becomes apparent that in simple bivariate comparisons (1) program types differ in their mean ratings, and (2) so do agency types, with programs in the more independent (as variously measured) agencies on average outperforming those inside ordinary executive departments, but that (3) independent agencies are more likely to have high-performing *types* of programs. This implies the need for a regression approach with appropriate controls. The result of the regression analysis shows an advantage persists for the independent agencies attributable to organization type, and this appears driven by higher ratings in particular types of programs administered by them. Most prominently, higher ratings for research programs are a key factor in the apparent organizational difference. Of course, the ratings themselves were the product of what was essentially an administrative experiment, yet to be repeated, and the number of observations for subtypes such as independent agency research programs is limited. Nevertheless, the overarching result of conditional independent agency advantage undercuts the more extreme criticisms of this form of executive governance. And the particular result with regard to government research suggests a line of further inquiry into whether institutionalized pluralism, involving leaders of diverse and independent views, facilitate success in programs designed to generate new knowledge.

## Theory

The current research appears to be the first study focusing on direct comparison of program performance across agency types of governance, but there is a basis to believe some differences in this area are possible and even likely. Previous analyses of organizational performance factors using the PART data have identified other aspects of agency leadership to have been important. Programs led by appointees associated with the presidential campaign have been found to underperform in comparison to those programs managed by other types of political appointees or by career executives (Gallo and Lewis, 2012). Positive effects on PART ratings have also been associated with a construct termed integrated leadership—the interconnections of leaders across different levels of the agency hierarchy (Fernandez et al., 2010). It therefore seems intuitively plausible that such important structural distinctions as having either collegial or unitary/hierarchical leadership, or having bipartisan rather than single-party leadership groups, could also have detectable effects. Indeed, both of these prior studies included an agency typology variable as part of their analysis, albeit as a control for the variables of primary interest. In Gallo and Lewis, independent commissions dropped out of significance in their later models, which relied only on the subset of those PART ratings considered credible by career executives (2012). Fernandez and his colleagues showed a non-significant but substantial in size (8 out a possible 100 points) positive effect of independent agencies on programs (2010). But it is noteworthy that their “independent agency” category included non-Departmental organizations such as the Environmental Protection Agency that have neither a multimember commission structure nor protection against presidential removal, and thus lack key structural features of independence.

However, the expectation of an effect does not indicate whether this effect is likely to be positive or negative. There is a line of thinking in political science which suggests, *ceteris paribus*, that the performance of independent agencies is likely to be relatively inferior, since their structure may represent interference by, and compromise with, opponents of the agency in Congress who have incentives to make it less effective (Moe, 1989, 1995). On similar grounds of traditional administrative principles, which associate expected efficiency to the clearest lines of authority and accountability, presidential reform commissions have assessed agencies insulated from presidential control as underperforming. The Brownlow Committee, reporting to Franklin Roosevelt in 1937, concluded that from a management perspective, “boards and commissions have turned out to be failures” (Brownlow, 1937, p. 30). Similarly, President Nixon's Ash Council proposed conversion of independent agencies into SHED form because: “The basic deficiencies we found are inherent in the collegial form itself, not in the number of commissioners” (Ash, 1971, p. 43). However, there was not a strong empirical basis for these assessments—which were, perhaps not coincidentally, in line with the interests of the Presidents who sought these reviews. Even with only qualitative information, it appeared that there were some agencies of both types which appeared to be performing poorly and others seemingly well-regarded (Robinson, 1971).

More recent theoretical approaches have by contrast suggested that collegial and bipartisan leadership could have advantages over single party hierarchies in avoiding problems of organizational “groupthink” (Sunstein, 2000). Ideologically homogenous opinions and the limited perspective of a single dominant personality could lead to misjudgments and agency underperformance. This problem would be most relevant when processes of internal group deliberation over uncertain phenomena are critical to agency decision making. In such circumstances, the presence of different policy views in agency leadership, as guaranteed by the usual bipartisan requirements, has been supposed to encourage more stable and centrist views, a result predicted from the social psychology of small deliberative groups (Breger and Edles, 2015, p. 2; Glaeser and Sunstein, 2009). Research conducted with groups of federal appellate judges sitting in three-person panels has shown less groupthink or “polarization” in the written opinions of bipartisan panels than in those of politically homogeneous ones (Sunstein et al., 2006). Similar comparative research has yet to be conducted for executive agencies, although Glaeser and Sunstein predicted the judicial result to generalize as an advantage for independent agencies (2009). Notably, the prediction is not of a global advantage for MICs in all activities of government, but one which is task-specific. This accords with the view expressed by administrative scholars in response to the recommendations of the Ash Council, which noted that Congressional practice and the qualitative evidence at that time suggested certain activities could be more suited to certain governance structures (Cramton, 1972).

Assuming this as a tentative hypothesis for what is at this stage exploratory research, the objective is to seek to characterize different governmental tasks and see how well they are performed in different types of agencies. The working theory would be that those tasks which could be enhanced by features associated with MICs, such as a broader representation of interests or alternative views on uncertain questions, would receive better performance scores. Other types of tasks, perhaps those involving decisiveness, speed, and clarity of direction, might show the opposite effect. In this way we are not assessing whether one agency or agency type is better, but the “fit” of the tasks performed by that agency to its structure. This requires a measure of agency type as the primary independent variable, interacting with program type, and affecting a measure of program performance as the primary dependent variable.

## Data Sources

As mentioned above, the source for performance-derived dependent variables in this analysis is the information collected by the Office of Management and Budget (OMB) during the George W. Bush Administration under the auspices of the Program Assessment Rating Tool (PART) (Moynihan, 2008). The PART was a government-wide initiative that lasted 7 years, until 2008, collecting data and creating a performance profile of the different programs administered by both SHEDs and MICs. Eventually OMB evaluated over 1000 programs constituting 98% of the federal budget, using a standard survey format (Moynihan, 2013). The four parts of the 25–30 question survey examined (1) program purpose and design (often having a statutory basis); (2) strategic planning and goal setting; (3) the management processes applied to the program, and (4) the results achieved by the program^2^ Each program was given a 0–100 score on each of the four subscales, and these sections were then weighted (20, 10, 20, 50%, respectively) to produce a total “PART score.” This score is the main dependent variable examined, but I also use the individual components of the score. Additional analyses use as the dependent variable the ordinal categories or “grades” by which OMB partitioned the PART scores: Ineffective (0–49), Adequate (50–69), Moderately Effective (70–84) Effective (85–100). Agencies could also receive, regardless of the overall score, a rating of Results Not Demonstrated (RND) if the program did not provide performance measures OMB found acceptable. Following the convention of Gallo and Lewis (2012), RND is inserted in the ordinal ranking between “Ineffective” and “Adequate^3^.”

Although PART data is used here in the absence of a superior alternative, its use comes with a number of caveats, and scholars have learned to treat its ratings with caution. As a performance management reform, PART was controversial during its implementation, and it still remains a subject of both analytical material and controversy among scholars of public administration (Kroll and Moynihan, 2021). OMB's ambition to compare different types of agencies with widely varying tasks and missions on a common measure—the attribute which makes it peculiarly useful in large-scale comparisons of the type attempted here—was criticized at the time and later for an inability to track “true” performance (Radin, 2006; Heinrich, 2012). A retrospective assessment concluded that while PART increased public transparency and encouraged agency changes to increase their scores, the Bush Administration's desire to tie lower scores directly to lower funding may have been an overreach, which created anxiety and resistance in the agencies being assessed (Hart and Newcomer, 2018). For instance, supporters of programs with poorly-rated performance often attribute their implementation challenges to inadequate resourcing, arguing for higher funding in response to lower scores—which was not OMB's usual perspective (Radin, 2006). However, as PART is still essentially unique in its comprehensiveness, it remains the basis for ongoing empirical research, especially with regard to the relation between program performance and later Congressional choices on funding or defunding programs (Han, 2020; Kasdin and McCann, 2022).

OMB attempted to collect program information in a neutral fashion, but a concern about bias raised regarding PART data has been that it was “politicized” in favor of programs more congenial to the conservative Bush Administration (Lavertu et al., 2013). What is unclear, however, is whether this effect arose from bias (unconscious or deliberate) or reflected something structural in either types of agencies or their leadership (Kroll and Moynihan, 2021). Clinton and Lewis rated government agencies as “conservative” or “liberal” based on a reputational survey, and found programs within more conservative agencies were rated more highly, and in particular, certain types of “redistributive” programs more common in “liberal” agencies, such as block grants, tended to be rated lower (Clinton and Lewis, 2008; Greitens and Joaquin, 2010). Controlling for these effects, programs led by political appointees, particularly former campaign staff, were outperformed by the relatively small number (<10%) of programs led by career civil servants (Lewis, 2008; Gallo and Lewis, 2012). An additional positive effect on PART “Results” (the fourth component of the PART score) was attributed to an index of “integrated leadership”—the sharing of key leadership roles across the hierarchy as measured by surveys of managers (Fernandez et al., 2010). These results, taken together, suggest an alternative interpretation for political difference might be that in “liberal” agencies overseen by a conservative administration, less cooperation occurred between political and career leaders, reducing the input of technical expertise and integrated leadership.

Even though the focus of this study is the effect of organizational type, the unit of analysis in the PART, and thus in this research, is the “federal program.” This is important to keep in mind since PART scores, and therefore those factors that raise or lower them, do not purport to assess *agencies* or their performance overall. Rather, they assess how well the programs OMB chose to examine operate within particular bureaucratic environments. For example, the Department of the Treasury was seen as successful in administering the public debt, but unsuccessful in managing a much smaller healthcare tax credit program. Consequently, it would be illegitimate to aggregate these scores in any obvious manner to infer that “Treasury” was performing worse (or better) than some other agency. The research question is whether there is statistical evidence that federal programs appear to be systematically benefited or hampered by the kind of organizational structure in which they are embedded.

The primary independent variable is either independence (an unavoidable double meaning) or commission structure, tested as alternatives. While closely overlapping, these are not the same; MICs have more of the characteristics of bureaucratic autonomy, but they vary, as do SHEDs, in their levels of independence (Datla and Revesz, 2012). The simplest partition, with commission status as an indicator variable, I take from the existing coding in the publicly available PART dataset constructed by Gallo and Lewis (2012). Supplemented with some additional variables, this dataset forms the basis for the analyses conducted here. Using their “commission” designation yields 75 programs associated with commissions, and 941 programs associated with other agencies (mainly Cabinet Departments) for 1016 PART rated programs in total. A tabulation (Table 1) comparing the OMB “grades” for these observations shows a clear difference between the two organizational types, with independent commissions having almost half their administered programs in the “Effective” category, as opposed to approximately one-sixth of the programs administered by departmental agencies. Table 1 also includes for reference the number of observations in each cell from the total dataset, which is used to calculate the percentage listed. (In parentheses are the observations used in some models requiring Selin ratings, discussed below; notably, no SHED programs rated “Effective” are lost in these models).

**Table 1:** Performance levels for two organizational categories.

**Category**	**SHED N (Selin)**	**SHED (%)**	**MIC N (Selin)**	**MIC (%)**
Ineffective	25 (24)	2.66	1 (1)	1.33
Res. Not Demo.	157 (154)	16.68	16 (14)	21.33
Adequate	288 (284)	30.61	10 (8)	13.33
Mod. Effective	315 (308)	33.48	11 (9)	14.67
*Effective*	156 (156)	16.58	37 (35)	49.33
Total	941 (926)	100.00	75 (67)	100.00

Independence as a variable is clustered and bimodally distributed around what has been referred to as MIC and SHED ideal types, but it is more complex than a mere yes/no indicator, and it would be analytically preferable in some ways to treat it as a continuous metric capable of incorporating the insight that organizational independence is, at least to some extent, a matter of degree rather than (or in addition to) being a matter of kind. Each of these different potential forms consists of a suite of features that imprecisely define it in the bureaucratic taxonomy, since not all entities under a particular label share identical aspects (Breger and Edles, 2015, p. 4–8; Datla and Revesz, 2012). For example, a small number of entities such as the Federal Energy Commission and Surface Transportation Board are distinctly appointed plural bodies embedded within the Departments of Energy and Transportation (Selin, 2015). The different legislative designs for agencies reflect choices not only on whether the organization is to be governed by a multimember group or a single administrator, but also the extent to which this leadership requires confirmation by the Senate, and if there are to be restrictions on presidential appointment and removal—particularly with regard to the partisan affiliation on who can serve and if they serve fixed terms terminable only for cause, or at the pleasure of the President (Lewis, 2004, p. 5). To these are added elements of statutory grants of organizational autonomy in budgeting (especially the capacity to self-finance apart from Congressional appropriation), litigation, and adjudication (Datla and Revesz, 2012; Kruly, 2013; Breger and Edles, 2015, p. 5).

Legal scholarship has tended to focus on the inability of the chief executive to remove agency officials absent a specified cause like misconduct or dereliction of duty (Breger and Edles, 2015, p. 160–161; Rao, 2013). Public administration, has, in addition to this aspect, tended to center on multiheaded governance and separation from the executive hierarchy, which, like removal limitations, diverge from traditional administrative theory (i.e., Brownlow, 1937, p. 4). Empiricists from political science and economics, however, focus on the effects of design choices on actually insulating the agency from political (especially presidential) control of policymaking, a notionally continuous rather than discrete classification (Gersen, 2010; Selin, 2015).

In order to more precisely measure what constitutes “independence,” Selin assembled a database of 321 agency and subagency structures, seeking to be comprehensive over the federal executive establishment and to reflect current organizations as contained in the 2013 United States Code (in contrast to the structure agencies had in their enacting statute, from which current structures often diverge) (2015)^4^. She then took fifty different organizational characteristics—none of which is actually labeled “independence”—and subjected them to a Bayesian latent variable procedure to identify the hidden unifying factor or factors that constitute operational independence. I include them here as alternative independent measures of agency typology.

The core result of her analysis was the identification of two main dimensions of agency independence, both of which are estimated as continuous variables. Her first dimension, “*Limits on the Appointment of Key Agency Decision Makers,”* directly relates to agency leadership and aligns with the traditional focus on the limitations imposed on the President with regard to appointment, removal and direction of the key officials (Selin, 2015). Appointment limitations most notably include the requirement that many MICs reflect explicit bipartisan membership, but also may involve requirements for expertise or geographic diversity. Her second dimension, “*Limitations on Political Review of Agency Policy Decisions*,” synthesizes a series of ex post facto mechanisms for the President and especially OMB to review or second-guess budgetary choices, litigation, legislative proposals and federal rulemaking (Selin, 2015). To the extent an agency need not consult or get permission for its actions, it has higher autonomy on this dimension.

Because the disaggregation of independence has the potential for greater precision in connecting structure to function, and because of the attractiveness of an external continuous measure of “independence” in place of simply noting the presence of a commission, Selin's metrics were evaluated as alternative independent variables for the present study and incorporated into the Gallo and Lewis PART dataset. Including Selin ratings reduces the number of observations to 993 total, with 67 attached to commissions. In order to attach her ratings, I used the lowest organizational level in the bureaucracy which she rated—thus for the National Institutes of Health's (NIH) programs for extramural research I use the rating for NIH, rather than the overall rating for the Department of Health and Human Services (HHS). HHS's rating is used as a catchall for programs not attached to a rated subagency in her database. The same protocol was employed with regard to the Forest Service vis-à-vis the Department of Agriculture, the Navy in relation the Department of Defense, and so on during the data merger process.

Key controls involve the types of programs administered, which are entered as a series of indicator variables from the coding in the dataset, itself based on OMB data (Gallo and Lewis, 2012). OMB's seven categories during PART were block or formula grants, grants subject to competition, capital acquisition activities (i.e., land purchase), credit programs, direct federal activities carried out by federal employees affecting the public, research (including external research grants), and regulatory programs (including rulemaking). Apart from the search for task-specific organizational advantages, if certain governmental tasks are inherently more difficult to manage (Radin, 2006 argues this is true for block grants for instance), it is important to control for this, both because it is an important extrastructural explanation of variation, and because program types are unevenly distributed between MICs and SHEDs. As Table 2 shows, proportions differ across organizational type, with independent commissions weighted toward overseeing regulatory activities, and to an extent, research, with traditional agencies oriented toward non-research grantmaking. As in Table 1, the number of observations included in models with Selin ratings is noted in Table 2 in parentheses; notably, no observations involving research programs are lost for either kind of agency. The percentages are for the full dataset. Because a number of independent agency subtypes have very few (<10) observations, generalization about these tasks based on organizational type is obviously very difficult.

**Table 2:** Types of programs administered by different organizations.

	**SHED (observations)**	**MIC (observations)**
Block grants	16.17% 152 (152)	4.00% 3 (2)
Competitive grants	18.30% 172 (171)	5.33% 4 (4)
Capital assets	8.51% 80 (74)	6.67% 5 (4)
Credit programs	3.93% 37 (37)	2.67% 2 (2)
Direct federal	35.21% 331 (324)	36.00% 27 (21)
Research and development	10.85% 102 (102)	14.67% 11 (11)
Regulatory	7.02% 66 (65)	30.67% 2 (23)

## Methodology and Results

As an initial approach, a straightforward analysis was conducted to compare the two types of organizations, using a two-sample *t*-test with unequal variances, against the total PART score with a range of 0–100^5^. As shown in the accompanying Table 3, there is a significant difference (*p* = 0.002) between these groups, with independent commissions showing a roughly 8 point higher PART score, consistent with Fernandez et al. (2010). Applying this same method to only the subset of 113 “research and development” programs administered by the two types of agencies produces an even stronger result, despite the limited number of observations for independent agencies. Commission-based research programs have a mean score of 90.4, while those in other agencies were rated on average at 73.4 (*p* < 0.001, two-tailed). This difference in R & D performance was an important driver of the apparent organizational effect but could not account for it completely. Performing a *t*-test on the rest of the dataset—i.e., all non-research programs—showed a remaining independent agency rating advantage of ~6 points (71.7 against an average SHED program rating of 65.5, *p* = 0.03, two-tailed).

**Table 3:** Performance of departmental agencies and independent commissions.

**Group**	**PART**	**Obs**.	**Std. Err**	**Std. Dev**.	**[95% Conf**.	**Interval]**
SHED	66.322	941	0.585	17.958	65.173	67.471
MIC	74.432	75	2.463	21.338	69.523	79.342
TOTAL	66.920	1,016	0.575	18.340	65.791	68.050

*t-test, t = −3.2025 p = 0.002 (two-tailed)*.

In order to test the basis and robustness of this organizational difference, ordinary least-squares regression analysis was conducted on the continuous dependent variable of the overall PART score, under a stepwise backwards elimination protocol. Further regressions were conducted separately on each of the four subcomponents of this program's score, program purpose, strategic planning, program management, and results/accountability. These are all continuous variables with a potential range of 0–100.

### First Study–The Main Effect of the Independent Agency

The first model builds off the bivariate comparison to add indicator (1 or 0) variables for each program type, but as controls. If multiple program types were listed, the first one in the Gallo and Lewis data was treated as primary and the source of the indicator. Although only explaining a relatively small portion of the data, this model continues to show in Table 4 a 5.65 point advantage of the commission structure. The effect of a commission is roughly equivalent to the score increase expected for a research program, and half the relative deficits associated with block or competitive grant programs, program effects which have received analysis in the literature on PART (Moynihan, 2013). As with the bivariate comparison, Model 1 is inconsistent with any simple view of commissions as inherently flawed.

**Table 4:** PART scores (OLS) by “commission,” model 1.

	**Coef**.	**Std. Err**.	**t**	**P>|t**	**[95%**	**Conf. Interval]**
Commission	5.652	2.112	2.68	0.008	1.506	9.798
Research	5.326	1.799	2.96	0.003	1.794	8.858
Block grant	−10.424	1.566	−6.65	0.000	−13.499	−7.351
Comp. Grant	−9.354	1.513	−6.18	0.000	−12.323	−6.386
Constant	69.193	0.7630	90.68	0.000	67.696	70.690

*R^2^ (adj) = 9.31%, F (4, 1011) = 27.04, p < 0.00005*.

In a second model, having assessed “commission,” I use an alternative specification for the “independent” variable in the form of Selin's Decision Maker autonomy measure. The results in Table 5 are similar and again show an independent agency advantage rather than a SHED managerial edge (because SelinDM ranges up to 2, a coefficient of roughly 2 is only slightly weaker than in Model 1). The main distinction is now that the entire remaining population is part of the calculation of independence. There is a very slight improvement in the explanation of variance (0.07%), but use of this measure also an implies that greater or lesser independence outside of commissions also has a (presumably positive) effect on program performance. In order to test this, I reran Model 2 only in the subgroup *without* commissions. In those circumstances, SelinDM becomes non-significant (with a slight negative coefficient). Her alternative measure of independence, from external political review, coded as SelinPR, when used as the independent variable, produces a non-significant but slight negative effect whether the model is estimated in the full group or the subgroup. The results of these specifications are consistent with the idea that structural form and plural leadership are more likely to be the drivers of organizational differences in this study than are agency insulation from accountability as such.

**Table 5:** PART scores (OLS) by Selin's decision maker, model 2.

	**Coef**.	**Std. Err**.	**t**	**P>|t**	**[95%**	**Conf. Interval]**
SelinDM	2.033	0.977	2.08	0.038	0.116	3.949
Research	5.095	1.812	2.81	0.005	1.538	8.651
Block grant	−10.511	1.595	−6.59	0.000	−13.641	−7.382
Comp. Grant	−9.423	1.546	−6.10	0.000	−12.457	−6.390
Constant	70.286	0.778	90.30	0.000	68.759	71.814

*R^2^ (adj) = 9.38%, F (4, 988) = 26.68, p < 0.00005*.

Nevertheless, there is also known variation in levels of autonomy *within* commissions, so I assessed if Selin's model could be useful in differentiating this group, and therefore identified as the new main variable of interest a hybrid between “Commission” and the (strongly correlated) Selin's Decision Maker axis. In this treatment, only MICs have an “independence score” (but it is no longer always 1). All SHEDs (whatever their original Selin score) have a zero for the predictor variable in Model 3. This variable therefore is intended to approximate and weight *independent* commissions by their degree of independence. As shown in Table 6, this is a notable improvement over Model 2–but not over Model 1–with the main variable now at *p* < 0.009 and a slightly higher level of explanation, *R*^2^ *(adj)*, than either Model 1 or Model 2.

**Table 6:** PART scores (OLS) by decision maker x commission, model 3.

	**Coef**.	**Std. Err**.	**t**	**P>|t**	**[95%**	**Conf. Interval]**
Independent commission	3.863	1.465	2.64	0.009	0.988	6.74
Research	5.246	1.809	2.90	0.004	1.695	8.796
Block grant	−10.670	1.580	−6.76	0.000	−13.769	−7.570
Comp. Grant	−9.591	1.526	−6.28	0.000	−12.585	−6.596
Constant	69.355	0.775	89.53	0.000	67.836	70.876

*R^2^ (adj) = 9.62%, F (4, 988) = 27.40, p < 0.00005*.

Because of the structure of the indicator variables, it is also feasible to estimate the partial effects of the independent commission as mediated through each particular kind of program. To do this, I generated interaction terms between Independent Commission (the independent variable specification from Model 3) and six of the program types (leaving one as a baseline), and this set of interaction terms now became the main variables of interest, and were subject to an elimination procedure. When this is done, it becomes apparent in Table 7 that the positive effects of independent commissions on PART scores are highly focused on two sets of programs, direct federal services and research programs, which is a superior explanatory Model 4 with *R*^2^ *(adj)* elevated over Model 3. The main effect of independent commission structure becomes non-significant in the presence of these interactions. Notably however, there are no significant *negative* interactions in this model to indicate areas where independent commissions are weak relative to SHEDs. The independent agency advantage therefore persists across multiple regression specifications and controls for differing proportion of hard-to-manage program types, and is consistent with apparent task-specific advantages of the kind Cramton predicted a half-century ago (1972).

**Table 7:** PART scores (OLS) by decision maker x commission, w/ interactions, model 4.

	**Coef**.	**Std. Err**.	**t**	**P>|t**	**[95%**	**Conf. Interval]**
Ind.com (research)	13.681	1.465	3.08	0.002	4.959	22.402
Ind.com (direct federal)	8.130	2.679	3.03	0.000	2.873	13.387
Research	3.969	1.883	2.11	0.035	1.695	8.796
Block grant	−10.600	1.572	−6.74	0.000	−13.684	−7.516
Comp. Grant	−9.593	1.516	−6.33	0.000	−12.568	−6.619
Constant	69.445	0.759	91.50	0.000	67.956	70.934

*R^2^ (adj) = 10.59%, F (5, 987) = 24.49, p < 0.00005*.

### Second Study–Specifying Effects on the Components of Organizational Performance

In order to explore further and partition the effects of structure on performance, I return to the simplest model of using a commission indicator variable, as its practical effects in this context operate so similarly to a Selin rating^6^, but alter the dependent variable from total PART scores to various alternatives. In addition to the type of program at issue, a number of known and potential effects on elements of the PART Score are entered as control variables, including the political orientation of the agency administering the program (taken from Clinton and Lewis, 2008), the total number of programs that agency administers, the period in which the OMB's evaluation occurred (early or late in the 2003 to 2008 history of PART), the presence of a career manager, and the size in budgetary terms of the program (Jung, 2013; Moynihan, 2013)^7^. Again ordinary least squares regression is used, with the aim of finding what sections of the score are impacted by the commission structure, in parallel with learning what types of programs are most affected.

Commission structure seemingly plays no role in the first components of the PART, as a commission variable is absent from these regression models. In addition to the late evaluation effect, the only factors affecting the purpose section of the PART are the indicator variables for type of program (Table 8A). Credit-offering programs perform worst, followed by block or grant formula programs, capital and acquisition operations and competitive grants. There is a possible minor negative effect for direct federal services (the only types of programs without negative coefficients are those with a regulatory or research focus), but only 3.8% (adjusted R^2^) of the variation in this PART component is explained by the factors collectively. The type of program at issue also affects strategic planning (Table 8B). Block and competitive grants, credit and direct federal programs all suffer a deficit, as do regulatory programs. In addition to the positive coefficient for late evaluation, a conservative political orientation and a larger budget also affect strategic planning positively.

**Table 8A:** Model for purpose and design section of PART.

**PART (1^**st**^ Sec.)**	**Coef**.	**Std. Err**.	**t**	**P>t**	**[95% Conf**.	**Interval]**
Late eval.	3.400	1.179	2.88	0.004	1.086	5.715
Capital assets	−4.186	2.447	−1.71	0.088	−8.989	0.617
Block grants	−9.376	1.921	−4.88	0.000	−13.145	−5.607
Comp. Grants	−3.956	1.893	−2.09	0.037	−7.671	−0.240
Credit	−12.321	3.204	−3.84	0.000	−18.610	−6.032
Direct federal	−2.414	1.633	−1.48	0.140	−5.620	0.791
Constant	90.126	1.365	65.99	0.000	87.446	92.806

*R^2^(adj) = 3.80%, F (6, 963) = 7.38 p < 0.00005*.

**Table 8B:** Model for strategic planning section of PART.

**PART (2^**nd**^ Sec.)**	**Coef**.	**Std. Err**.	**t**	**P>t**	**[95% Conf**.	**Interval]**
Budgetsize	1.723	0.383	4.50	0.000	0.971	2.474
Political	2.721	0.832	3.27	0.001	1.089	4.353
Late Eval.	5.773	1.504	3.84	0.000	2.821	8.725
Block grants	−14.314	2.556	−5.60	0.000	−19.329	−9.298
Comp. Grants	−6.901	2.576	−2.68	0.008	−11.955	−1.846
Credit	−10.333	4.092	−2.53	0.012	−18.364	−2.303
Direct federal	−4.388	2.079	−2.11	0.035	−8.468	−0.307
Regulatory	−8.811	3.163	−2.79	0.005	−15.018	−2.604
Constant	70.174	3.044	23.05	0.000	64.200	76.149

*R^2^(adj) = 10.59%, F (8, 961) = 7.38 p < 0.00005*.

Only with program management (Table 8C), does the leadership structure show even a possible effect, here with a positive coefficient, but only a non-significant *p-*value of 0.12. Although the political orientation of the agency does not affect the rating, a career manager can positively affect this component, and there is a positive effect of the number of programs the agency is overseeing. Grants programs, both block and competitive, have deleterious effects on management scores, although it should be emphasized that as with the model for program purpose, the model for management explains <4% of the variation.

**Table 8C:** Model for program management section of PART.

**PART (3^**rd**^ Sec.)**	**Coef**.	**Std. Err**.	**t**	**P>t**	**[95% Conf**.	**Interval]**
Commission	3.572	2.273	1.57	0.116	−0.889	8.032
Career app.	4.385	2.112	2.08	0.038	0.240	8.530
Late Eval.	4.093	1.130	3.62	0.000	1.876	6.310
# Programs	0.324	0.162	2.00	0.046	0.006	0.642
Block grants	−4.901	1.537	−3.19	0.001	−7.918	−1.885
Comp. Grants	−4.783	1.500	−3.19	0.001	−7.724	−1.842
Constant	81.819	1.060	77.18	0.000	79.739	83.900

*R^2^(adj) = 3.77%, F (6, 963) = 7.38 p < 0.00005*.

The effect of commissions becomes significant and powerful only for achieving actual program results (Table 8D) with *p*-value of 0.003 and positive coefficient of 9.5 (on a 1–100 scale). Career managers have a similar but slightly weaker effect. Research programs, those with larger budgets, and those in more conservative agencies also achieve higher results, while both types of grants programs continue to show negative effects for this PART component. Unlike the other three components, there is no effect of late evaluation.

**Table 8D:** Model for accountability/results section of PART.

**PART (4^**th**^ Sec.)**	**Coef**.	**Std. Err**.	**t**	**P>t**	**[95% Conf**.	**Interval]**
Commission	9.523	3.167	3.01	0.003	3.309	15.738
Budgetsize	2.311	0.405	5.71	0.000	1.518	3.105
Career app.	7.219	2.927	2.47	0.014	1.474	12.963
Political	3.733	0.884	4.22	0.000	1.997	5.468
Block grants	−10.045	2.358	−4.26	0.000	−14.672	−5.417
Comp. Grants	−6.930	2.389	−2.90	0.004	−11.618	−2.242
Research	9.764	2.532	3.86	0.000	4.796	14.733
Constant	36.621	2.863	12.79	0.000	31.003	42.238

*R^2^(adj) = 15.49%, F (7, 962) = 7.38 p < 0.00005*.

### Third Study–Independent Agency Effects on Ordinal Ratings in PART

This model, implemented *via* the “gologit2” command in Stata (Williams, 2006), allows separate estimation of the odds ratios for each level of the ordinal dependent variable, here the PART rating categories of Ineffective, Results Not Demonstrated, Adequate, Moderately Effective and Effective. That is, it reflects the likelihood of receiving the various levels of qualitative rating OMB assigned, and especially of achieving the top “Effective” rating, which was the ostensible goal of programs under the PART exercise (Moynihan, 2013). As is usual in logistic regression a coefficient >1 is indicative of a positive effect, and a coefficient below is indicative of a negative effect.

In Table 9 below, avoiding the “Ineffective” rating (and moving to the ambivalent status of “Results Not Demonstrated”) appears to be a function primarily of conservative political orientation and being evaluated late in the process. This is consistent with agencies (perhaps especially agencies in sympathy with OMB) learning how to respond sufficiently well to avoid a very poor rating. Late evaluation continues to be somewhat important to moving out of the two lowest categories into “Adequate,” as does the size of the program and possibly its characterization as research. Grant programs are more likely to be stuck in the lowest categories and so are independent commission programs, apparently because of the low number of MIC “Adequate” rated programs. The advantage of the independent commission starts to become apparent at the Moderately Effective level, where it has marginally significant positive effect (*p* = 0.052) with a coefficient of 1.736. Size, political orientation, career management, and being a research program, all also tilt the odds in favor of getting to this level, while block grants are again less likely to reach this rating.

**Table 9:** Generalized ordered logistic model of PART categories.

**Rating category**	**Odds ratio**	**Std. Err**.	**z**	**P>z**	**[95% Con**	**Interval]**
**Results N. D**.	**>**	**Level**	**0**			
Commission	0.8490	0.916	−0.15	0.879	0.102	7.032
Budget size	1.022	0.120	0.19	0.851	0.812	1.286
Career app.	0.564	0.424	−0.76	0.447	0.129	2.464
*Political*	*2.466*	*0.784*	*2.84*	*0.005*	1.32	4.599
*Late Eval*.	*4.480*	*2.830*	*2.37*	*0.018*	1.298	15.454
B. Grant	0.942	0.572	−0.10	0.922	0.287	3.099
Com. Grant	0.945	0.562	−0.09	0.925	0.295	3.029
Research	0.972	0.756	−0.04	0.971	0.212	4.462
constant	43.227	37.082	4.39	0.000	8.045	232.253
**Adequate**	**>**	**Levels**	**0–1**			
*Commission*	*0.501*	*0.157*	*−2.20*	*0.028*	0.271	0.927
*Budget size*	*1.252*	*0.060*	*4.67*	*0.000*	1.139	1.376
Career app.	0.992	0.338	−0.02	0.983	0.509	1.936
Political	1.113	0.108	1.11	0.268	0.921	1.346
*Late Eval*.	*1.368*	*0.243*	*1.76*	*0.078*	0.966	1.938
*B. Grant*	*0.361*	*0.0868*	*−4.24*	*0.000*	0.232	0.584
*Com. Grant*	*0.584*	*0.137*	*−2.29*	*0.022*	0.368	0.926
*Research*	*1.909*	*0.719*	*1.72*	*0.086*	0.912	3.995
Constant	1.33	0.438	0.87	0.386	0.698	2.54
**Mod.Effective**	**>**	**Levels**	**0–2**			
*Commission*	*1.736*	*0.493*	*1.94*	*0.052*	0.995	3.029
*Budget size*	*1.152*	*0.041*	*4.02*	*0.000*	1.075	1.235
*Career app*.	*1.661*	*0.439*	*1.92*	*0.055*	0.990	2.787
*Political*	*1.371*	*0.107*	*4.04*	*0.000*	1.176	1.597
Late Eval.	1.185	0.164	1.23	0.220	0.903	1.554
*B. Grant*	*0.597*	*0.123*	*−2.49*	*0.013*	0.398	0.896
Com. Grant	0.795	0.161	−1.13	0.257	0.534	1.183
*Research*	*2.838*	*0.692*	*4.28*	*0.000*	1.759	4.579
Constant	0.397	0.103	−3.56	0.000	0.239	0.660
**Effective**	**>**	**Levels**	**0–3**			
*Commission*	***5.377***	*1.504*	***6.01***	*0.000*	3.108	9.303
*Budget size*	*1.110*	*0.0484*	*2.40*	*0.016*	1.019	1.019
Career app.	1.566	0.451	1.56	0.119	0.890	2.753
*Political*	1.280	0.126	2.51	0.012	1.055	1.552
Late Eval.	1.209	0.207	1.11	0.267	0.865	1.690
B. Grant	0.720	0.212	−1.12	0.265	0.405	1.282
Com. Grant	0.752	0.226	−0.95	0.344	0.418	1.356
*Research*	1.739	0.429	2.24	0.025	1.072	2.819
Constant	0.094	0.031	−7.16	0.000	0.049	0.180

*Significant factors at different levels are in italics. Principal result in bold*.

The crucial effect appears at the final and highest rating category of “Effective.” The other factors that help a program reach the highest level are research focus, size, and a politically conservative host organization, but the role of independent commission-based programs stands out. At this level, the coefficient associated with independent commission programs is 5.377, meaning the odds ratio (“Effective/Less than “Effective”) of programs is more than 5 times higher for independent commissions^8^. This coefficient is not only much higher than any other for independent commissions; it far exceeds the coefficient of any other categorical variable in Table 9. Moreover, the z-score calculated for the commission coefficient at this level, 6.01, is larger than any other z-score in the model at any level. The independent commission's apparent capacity to frequently raise programs to the Effective level, achieved by driving higher external results, and a specific advantage in generating results in research or direct service programs, is the primary source of its overall advantage in PART scoring.

## Discussion

The assumption of some presidentially-oriented administrative reformers that executive departments would consistently outperform independent agencies is contradicted by the foregoing analysis of the PART data. This is not surprising, given the appearance of commission as a statistically significant positive factor in prior research (Fernandez et al., 2010; Gallo and Lewis, 2012), but it has been surprisingly understudied. The current analysis goes beyond past studies in showing more clearly the apparent basis of this advantage: the differential effect of commissions on administering research and direct federal programs, and the particular role this type of organization has in generating results and accountability (the fourth subcomponent of the PART) and in raising programs to the highest category of performance (“Effective”) in the ordered logistic model.

Mathematically, these last two features are intrinsically related. “Results” is not only the most heavily weighted, but also the most variable component, of the PART (s.d. 26.0), and the one with the lowest mean (50.0). The other components 1, 2, and 3 have means of 87.4, 76.9, and 83.7 and standard deviations of 18.3, 23.7 and 17.3, respectively. Consequently, the only practical way to get a top PART score is generally to drive higher results, and a factor associated with the former will have to be associated with the latter.

The rating of program purpose (Table 8A) was affected primarily by program type, and none of the organizational variables affected it all. This makes sense in that it is driven by the legislated structure of the program, which appears to set goals that may be intrinsically more ambiguous for certain types of government activities with few concrete outcomes. Strategic planning (Table 8B) is a fundamentally internal activity and the only program types without negative coefficients were capital/acquisitions programs and research programs, both of which share the trait of building upon past activities toward a goal and may be more amenable to long-range planning. Larger and more conservative (probably more “business-like”) programs also showed greater capacities for strategic planning.

Program management (Table 8C) involves primarily internal oversight, although some questions OMB asked refer to taking into account the views of external stakeholders in regulatory development, and of collaborating with related programs^9^. Collaboration and diverse perspectives present in commissions could play a limited role here and it would be useful in future research to examine the responses in this section at a more granular level, to see if independent commissions might have advantages when the questions involve engagement of a wider variety of stakeholders. The other predictors make intuitive sense in that career managers have a positive effect, and agencies with multiple programs develop some additional expertise in administering each one. Non-research grants programs, because of what is commonly a more tenuous legal relationship with the recipients, are harder to manage than those that involve supervision of direct federal employees or contractors (Radin, 2006).

With regard to program results and accountability, the independent commission advantage in operating externally becomes relevant and notable. Although the conservative agency advantage may be due to bias^10^, as some prior researchers have speculated, it also may be simply because the more businesslike “bottom line” perspective is more focused on quantifiable results. A larger program is also likely to have professionalized staff and processes more closely paralleling a corporation. The types of grant programs suffering a deficit are structurally problematic for results and accountability for the same reasons as they are difficult to manage–a diverse body of grantees is collectively responsible for result, usually in service delivery or financial assistance. This attenuates the capacity of agency leadership to engage in performance management. Research programs, on the other hand, could be evaluated by OMB on clear intermediate outcomes such as completed experiments and publications, and often have a more direct relationship between program staff and grant recipients than do services contracts involving state, local, or civil society entities as intermediaries (Moynihan, 2013).

Since these other effects make some sense, the question becomes what about independent commissions affects “Results” in a way that it affects no other section of the PART. The presence of a performance edge is a necessary but insufficient condition for showing organizational structure is responsible for the advantage. It seems unlikely, however, that political bias is responsible for the observed effects. Generally, the hypothesis would be that an organization could improve its scores by influencing the examiners (“working the ref”; Moynihan, 2013) if it was (1) politically aligned with the Bush Administration or (2) more structurally integrated with the White House. Both are untrue for commissions; for the 63 MIC programs with a politics score, their organizations are rated (non-significantly) less conservative (m = −1.35) than the Departmental scores associated with other programs (m = −1.05). Moreover, at the leadership level, these entities intrinsically had a significant number of Democrats. And as organizations legally outside the President's direct control, there is less likely to be a vested interest in protecting them or having programs within them tied to Presidential priorities. It therefore remains a plausible conclusion that it is something *structural* about independent commissions, rather than how they were evaluated, which explains their capacity to achieve high performance.

The analysis makes an effort to account for that fact independent commissions clearly differ from traditional agencies in the types of programs they oversee (as seen in Table 2 above), and that program types rated as less effective (notably “redistributive programs” and grants) are more prevalent in traditional agencies (Clinton and Lewis, 2008; Greitens and Joaquin, 2010). This is why program type is an absolutely necessary control variable in a study of this kind. More subtly, however, the preferential assignment by Congress of grants (for example) programs to SHEDs could reflect an underlying trend that the control would not fully capture: greater legislative care about expanding the scope and jurisdiction of a MIC. If this is so, when Congress is uncertain about where to place a new program–possibly in part because its objectives are ambiguous or it has been mainly enacted for political as opposed to performance purposes–it will more likely to go to a traditional agency like the Department of Health & Human Services with a very broad scope of activity. A bias in program assignment like this could theoretically lead to lower quality or harder-to-manage programs–of all program types–being more likely found in the SHEDs and account for difference, but this would require additional research and finding markers of program quality independent of the PART itself.

Another potential explanatory approach for the organizational type difference could be based on the idea that in MICs there would be a stronger level of internal demand for accountability by members of the non-Presidential party in leadership. The superior performance of Selin's Decision Maker axis (significant) to her Political Review axis (non-significant) does suggest that positive effects are indeed tied to leadership as opposed to other agency characteristics. But since the independent commissions differ in this index in several ways from departmental agencies (Lewis and Selin, 2013; Breger and Edles, 2015), the evidence developed here can only be suggestive in tying the observed effect to particular leadership features such as that of bipartisanship.

As an initial hypothesis, it might be supposed that this would primarily manifest itself in how the MIC acted externally, because unipartisan leadership may evince a form of “groupthink” that would tend to cause insularity and a divergence between the organization and those outside it (Glaeser and Sunstein, 2009). It could be hypothesized that any advantage due to viewpoint diversity would appear only in external operations under organizational control; this was principally the accountability and results portion of the PART and to lesser extent, the program management section. This is consistent with finding a strong and highly significant effect in the results section, a weaker effect in program management (in this case only a possible and non-significant effect), and no detectable effects in models predicting the other PART components. It is also consistent with finding commission effects mediated through positive effects on “direct federal” and “research” programs where the agency deals directly with, and may need to respond to, large stakeholder populations with differing perspectives.

Notably with research, the presence of multiple perspectives could be of value. The “MIC” driving the results here with regard to independence and research (and to some extent, the overall positive effect of the “research” variable on program performance) is the National Science Foundation (NSF). All but one of NSF's 11 rated programs were rated at the highest level of “Effective” and the other was rated just below at “Moderately Effective.” Although the 25-member governing board of NSF is not required to be bipartisan and is explicitly “apolitical,” its members are appointed in staggered 6-year terms by the President (and thus often Presidents of different parties), and these distinguished scientist-citizens from across the country are likely to represent more heterogenous views than organizations led by aligned political appointees and a hierarchical structure of single decisionmakers (whether career or political). However, whether the positive results should be attributed to the particular characteristics of the NSF as an organization *apart from its independence* cannot be determined from this data. Ideally, the performance of several independent agencies in administering research programs could be compared with that of several non-independent agencies, or alternatively, a more thorough causal case study could demonstrate how independence contributes to performance. But this must await future research, noting that this association is suggestive but not conclusive.

Another reason to be cautious about a direct relation between autonomy and research success is that the level of analysis used in this study is the program, and this is abstracted from how research performance is commonly evaluated. At the most concrete level, a particular piece of research (like this one, for instance) can be assessed in terms of its technical qualities, and its influence (as measured by citations, for instance), among other features. A research program, which is what the PART rated, presumably bears (or should bear) some relation to the success (in the sense just described) of the various research grants which comprise it. However, it seems in the case of PART to have understandably focused primarily on administrative characteristics rather looking underneath to aggregate how well the thousands of research grants the federal government gives out have performed, and which sets of research outperform others in relation to a defined benchmark.

This study then moves up one further level of abstraction and synthesizes the rated performance of programs seeking to identify significant factors that can distinguish *sets of programs* embedded in different institutions rather than research *per se*. To an extent, the analysis also reflects the next level up, as well, in assessing the overall research program performance of the federal government, although this comparison occurs not against other sponsors of research such as the private sector or other governments, but against other kinds of federal programs^11^. Put another way, the results here may suggest research programs do relatively well as programs, but tells us less how they do *as research*. Ultimately, any analysis of research performance must relate back to the productivity and significance of the new knowledge generated by the individual research teams, but the problems of composition and weighting are complex ones that I can only raise here.

One possible way to build on the agency type comparison would be to start from the bottom-up rather than the top-down, and evaluate the impact measures of publications produced by research grants from various federal research programs (Beacham et al., 2008). If appropriate controls are put in place, one would then systematically compare the aggregated impact measures associated with grants programs at independent agencies such as the NSF with those overseen by Departmental agencies such as the programs at the National Institutes of Health. Should this quite distinct approach to research program performance also yield a detectable independent agency advantage, it would provide support for the results found in the study, and more importantly, lay an evidentiary foundation for policy guidance. Such policy guidance, which is obviously premature as of yet, could mean more frequent assignment of research programs to commissions, or else adoption by other agencies of features such as more collegial and bipartisan governance.

Understanding we are operating on the organizational level, it worth pausing to consider whether other features of the MIC organization are plausible sources of its positive difference. A multimember leadership structure, with its blurring of lines of authority, does not seem intuitively conducive to greater accountability and drive for performance. Nor does the legal separation and protection from removal that are the hallmarks of “independence” (Selin, 2015) lead logically to the idea that agency leadership will be motivated to produce results, since their retention of office is very unlikely to be dependent on them. On the other hand, a potentially greater length and security of tenure in office could create more stability and sense of “ownership” in leaders that could be beneficial to performance (Heclo, 1977). Future research should seek to distinguish effects like this from any contribution bipartisanship may be making to performance.

If bipartisanship is indeed influential, it could provide an alternative explanation for the results of prior research on the PART. The value of a career manager is not simply or always greater management experience–many political appointees had more private sector and even public sector experience (Gallo and Lewis, 2012). They also offer a different perspective that may be lacking in a leadership structure that is otherwise politically homogenous, either adding political diversity or just a nonpolitical viewpoint. Similarly, Fernandez et al. (2010) discovered that the construct of “integrated leadership” has a positive effect on the PART, which could be due to the overcoming of insularity in some organizations by reaching and incorporating more intellectually diverse career staff views into the process of program management. If this interpretation has some validity, the performance advantages of bipartisan commissions would not be a *distinct* effect on performance, but one of a species of related effects, centered around the differential capacity of some government organizations to incorporate a wider expanse of expertise and ideas than others (see Robinson, 1971 for a precursor of this argument in reaction to the Ash Council Report).

Further investigation on the social dynamics in agency leadership phenomenon is required to support a groupthink avoidance explanation of the commission advantage, either in research programs or in other governmental tasks. However, the existence of this advantage, and its substantial apparent level of effect in driving federal programs toward excellence, is of importance standing alone, and worthy of sustained further study. Ultimately future research along these lines will seek to better understand what types of programs should be assigned to what type of organization, matching function to structure in ways that optimize agency performance.

## Data Availability Statement

Publicly available datasets were analyzed in this study. This data can be found at: https://my.vanderbilt.edu/davidlewis/data/.

## Author Contributions

The author confirms being the sole contributor of this work and has approved it for publication.

## Conflict of Interest

The author declares that the research was conducted in the absence of any commercial or financial relationships that could be construed as a potential conflict of interest.

## Publisher's Note

All claims expressed in this article are solely those of the authors and do not necessarily represent those of their affiliated organizations, or those of the publisher, the editors and the reviewers. Any product that may be evaluated in this article, or claim that may be made by its manufacturer, is not guaranteed or endorsed by the publisher.

## Footnotes

^1^As a Deputy Assistant Secretary in this Administration, I had a peripheral role determining one of the 1016 ratings of federal programs in the PART, but inclusion of this data point does not affect the principal results discussed here.

^2^Available online at: http://georgewbush-whitehouse.archives.gov/omb/expectmore/part.html.

^3^The basis for this ranking is not discussed, and is not part of the OMB ranking; however it follows intuitively from the idea that the worst result for a program would be “Ineffective” but that any “passing grade” such as “Adequate” would be better than “Results Not Demonstrated.” The mean PART score for RND programs is 40.5 (although some can be substantially higher), which is higher than the mean for “Ineffective” programs (m = 36.7), but much less than the mean for “Adequate” (59.5). At the suggestion of an anonymous reviewer, I conducted basic two-sample t-test comparisons without the RND programs, and they do not change the main results – the overall advantage for independent commissions in mean program score increases from 8 to 10 points, and task-specific commission advantage with regard to research program performance falls from 17 to 14 points. Both differences remain highly significant with or without the RND-rated programs.

^4^Because the agency structure was assessed several years after OMB assessed program operations (2003–2008), there may be some divergence or anachronism in particular cases if their statutory structure was altered during the first term of the Obama Administration.

^5^The samples are substantially different in size and the equal variance assumption was tested using Bartlett's test for equal variances: chi(sq) = 4.4849, Prob = 0.034. The test is violated at the 0.05 level, and therefore the unequal variance correction was applied in Stata.

^6^A comparison of Model 1 and Model 3 shows roughly equivalent *p*-values, with a slight edge (0.008–0.009) given to the commission indicator variable in Model 1 (which has a larger number of observations).

^7^This is entered as the log of the budget for the years between 2007 and 2009 (following Gallo and Lewis, 2012). Use of the non-logged budgetary figures was found to produce nonsignificant results.

^8^This largely formalizes the results from Table 1, the breakdown of grades by organizational type: calculation from these statistics shows (Effective/Less than Effective) ratios of 37/38 for MICs (.97) and 156/785 (.20) for SHEDs, which yields a rough odds ratio calculation of 4.9 between them.

^9^For example, see the typical questions issued to the Animal Plant and Health Inspection Service, http://www.aphis.usda.gov/library/manuals/APHIS1220/tab6omb.pdf.

^10^It is interesting to note that a late evaluation does *not* affect the “results” portion of the PART score, although it affects the score on program purpose, strategic planning and program management. This tends to favor the view that the late evaluation effect is based on programs adapting to the PART process rather than to a desire by OMB to show performance improvements. Agencies could not easily alter the results of their programs in the way they could clarify goals, make strategic plans, and adopt management processes. But in this model, there was no evidence a later OMB “graded easier” in its demand for performance results, the most important element of the PART score.

^11^It would be possible to move up further in levels of analysis, to assess the overall performance of the United States in generating research, in comparison with other national systems, or even the global performance of research programs, assessed and compared across time.
